# Cytochrome P450 2E1 (CYP2E1) regulates the response to oxidative stress and migration of breast cancer cells

**DOI:** 10.1186/bcr3574

**Published:** 2013-11-08

**Authors:** Travis Leung, Ramkumar Rajendran, Subir Singh, Richa Garva, Marija Krstic-Demonacos, Constantinos Demonacos

**Affiliations:** 1 School of Pharmacy and Pharmaceutical Sciences, Stopford Building, University of Manchester, Oxford Road, Manchester M13 9PT, UK; 2 Faculty of Life Sciences, Michael Smith Building, University of Manchester, Oxford Road, Manchester M13 9PT, UK; 3 School of Environment & Life Sciences, College of Science & Technology, Cockcroft Building, University of Salford, The Crescent, Salford M5 4WT, UK

## Abstract

**Introduction:**

The cytochrome P450 (CYP) enzymes are a class of heme-containing enzymes involved in phase I metabolism of a large number of xenobiotics. The CYP family member CYP2E1 metabolises many xenobiotics and pro-carcinogens, it is not just expressed in the liver but also in many other tissues such as the kidney, the lung, the brain, the gastrointestinal tract and the breast tissue. It is induced in several pathological conditions including cancer, obesity, and type II diabetes implying that this enzyme is implicated in other biological processes beyond its role in phase I metabolism. Despite the detailed description of the role of CYP2E1 in the liver, its functions in other tissues have not been extensively studied. In this study, we investigated the functional significance of CYP2E1 in breast carcinogenesis.

**Methods:**

Cellular levels of reactive oxygen species (ROS) were measured by H_2_DCFDA (2 2.9.2 2′,7′-dichlorodihydrofluorescein diacetate) staining and autophagy was assessed by tracing the cellular levels of autophagy markers using western blot assays. The endoplasmic reticulum stress and the unfolded protein response (UPR) were detected by luciferase assays reflecting the splicing of mRNA encoding the X-box binding protein 1 (XBP1) transcription factor and cell migration was evaluated using the scratch wound assay. Gene expression was recorded with standard transcription assays including luciferase reporter and chromatin immunoprecipitation.

**Results:**

Ectopic expression of CYP2E1 induced ROS generation, affected autophagy, stimulated endoplasmic reticulum stress and inhibited migration in breast cancer cells with different metastatic potential and p53 status. Furthermore, evidence is presented indicating that *CYP2E1* gene expression is under the transcriptional control of the p53 tumor suppressor.

**Conclusions:**

These results support the notion that CYP2E1 exerts an important role in mammary carcinogenesis, provide a potential link between ethanol metabolism and breast cancer and suggest that progression, and metastasis, of advanced stages of breast cancer can be modulated by induction of CYP2E1 activity.

## Introduction

Cytochrome P450 (CYP450) is a superfamily of hemoproteins essential for the biotransformation of drugs [1]. They are mainly localised in the liver, participating in the phase I metabolism of a wide range of exogenous compounds and the biosynthesis and metabolism of endogenous hormones [2]. Apart from the liver, CYPs are also expressed in other tissues such as lung, kidney and hematopoietic tissue [3], and specific isoenzymes of the superfamily have been identified in tumours [4] where they are suggested to affect the response to anticancer therapy [4,5]. CYP450s are highly conserved across species implying that, in addition to their function in the metabolism of xenobiotics, these enzymes possibly exert broader physiological functions [6]. Consistent with this view, the CYP2E1 isoenzyme has been implicated in a variety of pathological conditions such as diabetes, non-alcoholic steatohepatitis (NASH) and cancer, possibly as a result of its capacity to produce high levels of reactive oxygen species (ROS) [7].

CYP2E1 metabolizes several small molecules such as ethanol, acetaminophen and pro-carcinogens like nitrosamines and azo compounds [3]. CYP2E1-mediated metabolism of these compounds generates toxic intermediates and excessive amounts of ROS [7]. High ROS levels, and hence oxidative stress due to increased CYP2E1 protein levels and induced enzymatic activity, are the main causes of various liver diseases associated with chronic alcohol consumption [8] and a variety of other pathophysiological conditions including diabetes type II and obesity [9].

Since CYP2E1 is a key determinant of the cellular redox state generating free radicals in a non-specific manner, even in the absence of a substrate the gene expression of this enzyme is tightly regulated [10]. Indeed, links between CYP2E1 protein levels and cytokines activity have been shown in recent reports [11] as well as variable CYP2E1 gene expression in numerous inflammatory diseases including cancer [12,13]. Autophagy is one of the pathways induced by elevated ROS levels which triggers the accumulation of various autophagy-regulated genes (ATGs) including beclin-1 and the light chain 3 (LC3) [14], thereby stimulating the formation of the autophagosome in cancer [15,16].

Furthermore, oxidative stress and other cellular tensions, such as DNA damage and viral infection, impair the protein-folding process resulting in the accumulation of misfolded proteins within the endoplasmic reticulum (ER) lumen [17], stimulating the initiation of the unfolded protein response (UPR) [18]. UPR takes place in the ER lumen and is a major signal transduction pathway aiming to alleviate ER stress by removing accumulated unfolded proteins from this cellular compartment [18].

Clinical studies have indicated that stage I breast tumours express higher CYP2E1 mRNA levels compared to stages II, III and IV [19]. Taken together, the differential expression of CYP2E1 in different tumours and various stages of breast cancer, with its capacity to induce ROS production [7], raises the questions whether CYP2E1 cellular levels could be an indicator of breast cancer progression and which are the factors involved in its differential regulation of gene expression in the various stages of breast cancer. Here we present evidence to suggest that ectopically expressed CYP2E1-mediated oxidative stress regulates autophagy, ER stress and migratory potential and its gene expression is regulated by the p53 tumour suppressor in a cell-type-dependent manner in breast cancer cells.

## Methods

### Cell lines, cell culture and constructs

The human breast carcinoma cell lines MCF7 (p53+/+) [20], T47D (mutated p53) [20], MDA-MB-231 (mutated p53) [20,21] and MDA-MB-157 (p53-/-) [20] (obtained from the European Collection of Cell Cultures (ECACC)) were maintained in Dulbecco’s modified Eagle’s medium (Sigma-Aldrich, Gillingham, UK), supplemented with 10% foetal bovine serum (Gibco, Paisley, UK) and 1% penicillin/streptomycin (Lonza, Allendale, NJ, USA) at 37°C in a humidified atmosphere containing 5% CO_2_. Cells were treated with 10 μM etoposide (Sigma-Aldrich) for 16 hours, 500 μM N-acetylcysteine (NAC) (Sigma-Aldrich) for 16 hours, 2.5 mM acetaminophen (APAP) (Sigma-Aldrich) for 3 h, 20 μM chlormethiazole (CMZ) (Sigma-Aldrich) for 16 h, 1 μM bortezomib (Bort) (Selleckchem, Stratech Scientific Ltd., Newmarket, UK) the MCF7 for 24 and the MDA-MB-231 cells for 8 h and 100 mM ethanol for either 24 or 2 h as indicated in the figure legends. Transient transfections were carried out using the polyfect transfection reagent (Qiagen, Crawley, UK), according to the manufacturer’s instructions. Constructs used for ectopic expression included PCDNA3 [22,23], and β-galactosidase [22,23]. Human CYP2E1 luciferase reporter containing putative p53 binding sites was constructed by amplifying the upstream region of the CYP2E1 promoter -7873 to -5896 (counted from the translation initiation site) and inserting it in the pGL3 promoter luciferase vector (Promega, Madison, WI, USA). CYP2E1 short hairpin RNA (shRNA) P-silencer was synthesised using the P-silencer 2.1-U6 hygro vector (Agilent Technologies, Wokingham, UK). Dr. Cederbaum (Mount Sinai School of Medicine, New York) kindly provided the CYP2E1 cDNA. The pCAX-HA-2xXBP1deltaDBD9anATG)-Luc-F luciferase reporter [24] is a generous gift from Dr. Iwawaki (Frontier Research System, Riken, Japan).

### Immunoblotting and antibodies

High-salt lysis buffer (50 mM Tris-HCl pH 7.5, 400 mM NaCl, 5 mM ethylenediaminetetraacetic acid (EDTA) pH 8, 0.5% NP-40, 1% Triton X-100, 1 mM dichlorodiphenyltrichloroethane (DDT), 1 mM phenylmethylsulphonyl fluoride (PMSF) and 1 μg/ml protease inhibitor cocktail (pepstatin, aprotinin, and leupeptin)) was used to harvest cells. After SDS-PAGE and electroblotting membranes were incubated with anti-CYP2E1 (Abcam, Cambridge, UK, ab28146), anti-p53 (Santa Cruz Biotechnology, Santa Cruz, CA, USA, sc-126), anti-β-actin (Abcam, 8227), anti-LC-3 (Cell Signaling Technologies, Beverly, MA, USA, 4108S), anti-beclin-1 (Cell Signaling Technologies, 3495S), anti-Atg5 (Cell Signaling Technologies, 2630S), anti-Atg7 (Cell Signaling Technologies, 2631S), anti-CCAAT/enhancer-binding protein (C/EBP) homologous protein (CHOP) (Cell Signaling Technologies, 2895S), and anti-GRP78 (Santa Cruz Biotechnology, sc-376768) antibodies.

### 2 2.9.2 2′,7′-dichlorodihydrofluorescein diacetate (H_2_DCFDA) staining

Cells were transiently transfected with the indicated constructs and 16 h after transfection dissociated from the plates and centrifuged at 1,200 rpm for 3 mins. Cells were then incubated with 1 ml of APC-H7-conjugated CD20 antibody (BD Biosciences, Franklin Lakes, NJ, USA, 641396) where indicated, washed three times with PBS, incubated with H_2_DCFDA (Invitrogen, Carlsbad, CA, USA, D399) in the dark at 37°C for 30 min and subjected to fluorescence-activated cell sorting (FACS) analysis using CYAN-ADP flow cytometer (Dako, Glostrup, Denmark) following the fluorescence profile of 2 2.9.2 2′,7′-dichlorodihydrofluorescein diacetate (H_2_DCFDA) and APC-H7 probes.

### Chromatin immunoprecipitation

The process for chromatin immunoprecipitation (ChIP) has been described in the past [22,23]. Briefly chromatin was cross-linked with 1.42% formaldehyde and cross-linking was quenched by addition of 125 mM glycine. Cells were then harvested in IP buffer (50 mM Tris-HCl pH 7.5, 150 mM NaCl, 5 mM EDTA pH 8, 0.5% NP-40, 1% Triton X-100, 1 mM DDT, 1 mM PMSF, 1 μg/ml protease inhibitor cocktail (pepstatin, aprotinin, and leupeptin), 20 mM β-glycerol phosphate, and 2 mM sodium orthovanadate). Chromatin was sheared by sonication (Bioruptor, Denville, NJ, USA) and subjected to immunoprecipitation with the indicated antibodies. Precipitated DNA fragments were then amplified in PCR reactions with specific primers (F’ GAGGAGAGGCAAGTTTG and R’ AGTCCCTTCGCCTGTTTCTT) flanking the putative p53 binding sites identified within the *CYP2E1* promoter and analysed by agarose gel electrophoresis.

### UPR study using pCAX-HA-2xXBP1deltaDBD9anATG)-Luc-F construct

The principle of the pCAX-HA-2xXBP1deltaDBD9anATG)-Luc-F function has been described by Iwawaki and Akai [24]. In brief, stimulation of the ER stress in cells upregulates UPR by activating the ER signalling proteins PKR-like endoplasmic reticulum kinase (PERK), activating transcription factor 6 (ATF6) and inositol-requiring enzyme 1 (IRE1). Upon activation, the endoribonuclease activity of IRE1 catalyses splicing and removal of an intron from the Xbox binding protein 1 (XBP1) encoding mRNA thereby allowing its translation into a functional transcription factor [18]. Initiation of UPR and activation of XBP1 induces luciferase production from the pCAX-HA-2xXBP1deltaDBD9anATG)-Luc-F reporter whereas in the absence of UPR the inactive XBP1 is unable to stimulate luciferase production [24].

### Scratch wound cell migration assay

The scratch wound assay was performed as described previously [25]. Briefly coverslips were placed in 6-well plates and an insert (ibidi, Munich, Germany) was placed onto the coverslip before cells were seeded. After ibidi chambers adhered onto the coverslips, 3 × 10^4^ of MCF7, 3 × 10^4^ of MDA-MB-231 and 6 × 10^4^ of MDA-MB-157 cells were seeded into each side of the chamber and incubated overnight at 37°C to subclonfluent stage and then transiently transfected or treated with different drugs as indicated. The inserts were then removed from the coverslips, 2 ml of cell culture medium was added and cells were incubated at 37°C to allow cell migration for 16 h for MCF7 and MDA-MB-157, and 8 h for MDA-MB-231 cells. Subsequent to cell migration, cells were fixed with 4% paraformaldehyde in PBS for 30 min at room temperature. After cell fixation, cells were washed three times with PBS and permeabilised with Triton (X-100). Anti-β-actin antibody (Abcam, ab8227) and 4′,6-diamidino-2-phenylindole (DAPI) (Sigma-Aldrich, F6057) were used to stain cells.

No patients’ cells/tissues or animals were used in this study therefore there was no need for ethical approval.

### Statistical analysis

Statistical analysis of differences was carried out using Student’s *t* test, one-way analysis of variance (ANOVA) and Tukey’s *post hoc* test. Values *P* <0.05 are indicated with two asterisks and *P* <0.01 with three asterisks.

## Results

### CYP2E1 contributes to ROS generation in breast cancer cells

It has been shown that CYP2E1 is one of the most active CYP450 isoforms in generating intracellular ROS [26] and oxygen radicals are associated with cancer development and metastasis [27]. Several observations link CYP2E1 with inflammatory reactions and carcinogenesis in different tissues [28,29] suggesting that CYP2E1 might be involved in the regulation of tumour growth [29]. To investigate the role of CYP2E1 in breast cancer, ROS generation was monitored in MCF7, and MDA-MB-157 cells treated with ethanol, which is a known CYP2E1 inducer. In addition, ROS levels were monitored in MCF7, MDA-MB-231 and MDA-MB-157 cells treated with either the topoisomerase II inhibitor etoposide, which induces the transcriptional activity of transcription factors such as p53 and NRF2 which could potentially be involved in the regulation of CYP2E1 gene expression [30,31], the anti-oxidant NAC or ectopically expressing CYP2E1.

**Figure 1:**
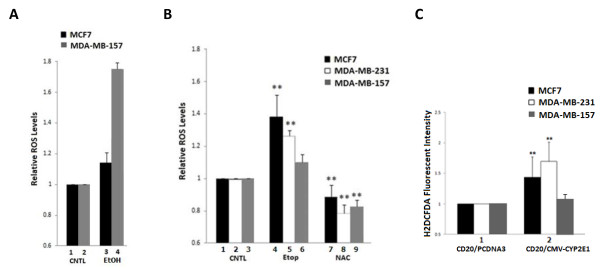
**Ectopic expression of CYP2E1 induces ROS generation in breast cancer cells. (A)** MCF7 and MDA-MB-157 cells were treated with ethanol and ROS levels were estimated using H_2_DCFDA and FACS analysis. **(B)** MCF7, MDA-MB-231 and MDA-MB-157 cells were treated with etoposide and NAC and ROS levels were estimated as in **(A)**. **(C)** MCF7, MDA-MB-231 and MDA-MB-157 cells were co-transfected with CD20 and either PCDNA3 or CMV-CYP2E1 expression plasmid. Subsequent to transfection, cells were treated with H_2_DCFDA fluorescent stain, and transfected cells were sorted with an APC-conjugated anti-CD20 antibody and FACS analysis. Data represent fold induction of ROS levels. Error bars indicate standard deviation obtained from three independent experiments performed in duplicates. Asterisks indicate significant difference at *P* <0.05. CYP2E1, cytochrome P450 E1; FACS, fluorescence-activated cell sorting; H_2_DCFDA, 2 2.9.2 2′,7′-dichlorodihydrofluorescein diacetate; NAC, N-acetylcysteine; ROS, reactive oxygen species.

Elevated intracellular ROS levels were observed in ethanol-treated MCF7 and MDA-MB-157 cells (Figure 1A, compare bar 3 to bar 1 and bar 4 to bar 2 respectively). Increased ROS levels were also recorded in MCF7 and MDA-MB-231 cells treated with etoposide (Figure 1B, compare bar 4 to bar 1 and bar 5 to bar 2 respectively). Moderate increase of ROS levels was observed in MDA-MB-157 cells treated with etoposide (Figure 1B, compare bar 6 to bar 3). Decreased ROS levels were identified in MCF7, MDA-MB-231 and MDA-MB-157 cells treated with the antioxidant reagent NAC (Figure 1B, compare bars 7, 8 and 9 to bars 1, 2 and 3 respectively). Increased intracellular ROS levels were also detected in MCF7 and MDA-MB-231 cells overexpressing CYP2E1 (Figure 1C, compare black and white bars 2 to black and white bars 1 respectively). Overexpression of CYP2E1 did not significantly affect the ROS levels in the MBA-MD-157 cells (Figure 1C, compare grey bar 2 to grey bar 1).

ROS levels detected by FACS analysis in MCF7, MDA-MB-231 and MDA-MB-157 cells were a result of CYP2E1 ectopic overexpression as cells transiently transfected with CYP2E1 expression vector were selected by CD20 co-transfection as described in the Materials and methods.

### Role of CYP2E1 in autophagy

To shed light on the role of the CYP2E1-mediated ROS generation in breast cancer [14,15] the levels of the autophagic markers beclin-1, LC3, Atg5 and Atg7 were monitored in MCF7 and MDA-MB-231 breast cancer cells transiently transfected with CYP2E1 expression vector or shRNA to silence the CYP2E1 expression.

**Figure 2:**
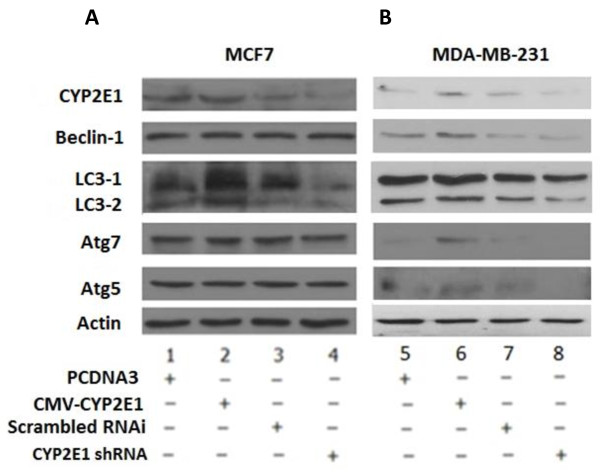
**CYP2E1 is involved in orchestrating autophagy in breast cancer.** MCF7 **(A)** and MDA-MB-231 **(B)** breast cancer cells were analysed for the autophagy biomarkers beclin-1, LC-3, Atg7 and Atg5 protein levels. Cells were transfected with PCDNA3, CMV-CYP2E1 expression plasmid, scrambled RNAi, and CYP2E1 shRNA and total cell extracts were subjected to western blot analysis. One representative from three independent experiments is shown. CYP2E1, cytochrome P450 E1; LC-3, light chain 3; shRNA, short hairpin RNA.

CYP2E1 ectopic expression in MCF7 cells shown in Figure 2A (compare lane 2 to lane 1) and CYP2E1 silencing by CYP2E1 shRNA (Figure 2A, compare lane 4 to lanes 1 and 3) did not have any significant effect on beclin-1, LC3-2/LC3-1 ratio, Atg5 and Atg7 protein levels in these cells (Figure 2A, compare lane 2 to lanes 1 and 3). On the other hand increased beclin-1, LC3-2/LC3-1 ratio, Atg5 and Atg7 protein levels coincided with CYP2E1 ectopic expression in MDA-MB-231 cells (Figure 2B, compare lane 2 to lanes 1 and 3). Reduced protein levels of these autophagy markers were observed in MDA-MB-231 cells transfected with shRNA against CYP2E1 (Figure 2B, compare lane 4 to lanes 1 and 3). Western blot analysis of the autophagic markers in MDA-MB-231 cells lent support to the notion that autophagy is regulated by CYP2E1 in MDA-MB-231 breast cancer cells.

### Role of CYP2E1 in the regulation of the endoplasmic reticulum stress

Recently published observations have indicated that CYP2E1 is mainly localised within the endoplasmic reticulum and functions as metabolic enzyme by oxidising xenobiotics [32]. Due to their highly reactive properties, ROS have a short half-life and limited diffusion distance [33], therefore, they usually inflict cellular damage and impact molecular pathways only near the site of their production [34]. It is well known that generation of ROS, sensitisation of ER and initiation of the unfolded protein response [35] and autophagy are closely related processes [36,37]. Taken together all the above information triggered our interest to explore the potential involvement of CYP2E1 in the regulation of ER stress.

**Figure 3:**
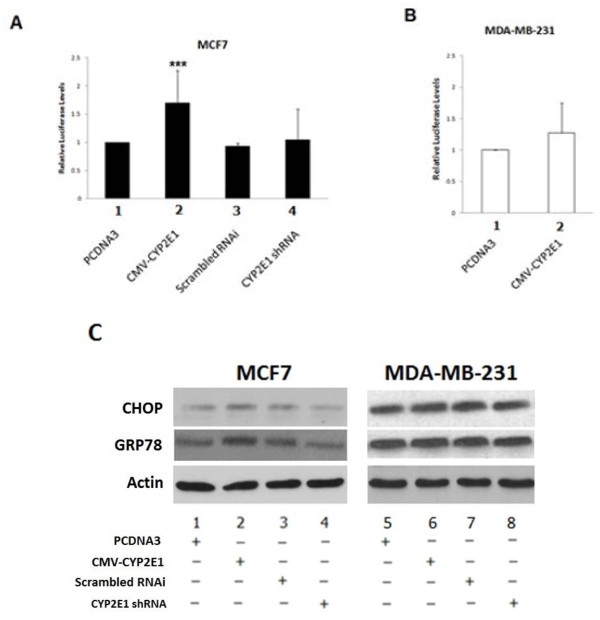
**ER stress is induced in MCF-7 and MDA-MB-231 breast cancer cells ectopically expressing CYP2E1. (A)** MCF7 and **(B)** MDA-MB-231 breast cancer cell lines were transfected with ERAI-Luc and indicated constructs. Cells were harvested and luciferase activity was measured. Luciferase readings were normalized to the β-galactosidase. Data are representative of three independent experiments. Error bars represent standard deviation. **(C)** MCF7 and MDA-MB-231 breast cancer cell lines were transfected with the indicated constructs and submitted to western blot analysis for the ER stress markers CHOP and GRP78. One representative from three independent experiments is shown. CHOP, CAATienhancer-binding protein homologous protein; CYP2E1, cytochrome P450 E1; ER, endoplasmic reticulum; GRP78, glucose-regulated protein 78.

The CYP2E1-mediated ER stress and UPR were assessed in breast cancer cells by monitoring the luciferase activity of the pCAX-HA-2xXBP1deltaDBD9anATG)-Luc-F reporter in breast cancer cell lines transfected with PCDNA3, CYP2E1, scrambled RNAi, or CYP2E1 shRNA. Increased XBP1 splicing was observed in MCF7 cells overexpressing CYP2E1 (Figure 3A, compare bar 2 to bar 1) whereas silencing of CYP2E1 expression by CYP2E1 shRNA transfection resulted in pCAX-HA-2xXBP1deltaDBD9anATG)-Luc-F activity similar to that exhibited by the cells transfected with empty vector or scrambled RNAi (Figure 3A, compare bar 4 with bars 1 and 3). MDA-MB-231 cells overexpressing CYP2E1 on the other hand, displayed a slight not significant increase of XBP1 splicing (Figure 3B, compare bar 2 to bar 1) [24]. Evaluation of the ER stress markers CHOP and GRP78 indicated increased levels of these proteins in the MCF7 cells transfected with CYP2E1 expression vector compared to MCF7 cells in which CYP2E1 expression had been silenced (Figure 3C, compare lane 2 to lanes 1 and 3 and lane 4 to lanes 1 and 3 respectively). No changes of CHOP and glucose-regulated protein 78 (GRP78) protein levels were observed in MDA-MB-231 cells (Figure 3C, lanes 5 to 8).

### CYP2E1 modulates cell migration potential

Results shown in Figure 1 provide evidence that CYP2E1 is a potential regulator of intracellular ROS levels in breast cancer cells. The link between oxidative stress and metastasis has been demonstrated in several recent publications [38,39]. Taken together the role of CYP2E1 in inducing ROS generation and the fact that CYP2E1 is differentially expressed in early rather than later stages of breast cancer [19] implies that this cytochrome P450 isoenzyme might regulate migration of breast cancer cells. To understand the role of CYP2E1 in these processes, the low invasive MCF7 and the highly invasive MDA-MB-231 as well as the MDA-MB-157 breast cancer cells were transiently transfected with CYP2E1 expressing constructs or vectors silencing the expression of this enzyme and cell migration was analysed employing the scratch wound assay.

**Figure 4:**
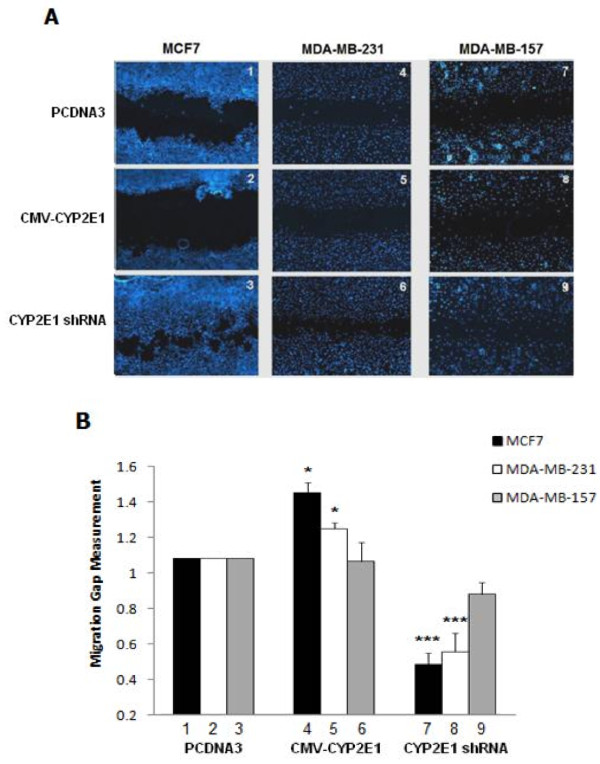
**Ectopic expression of CYP2E1 regulates migration of breast cancer cells. (A)** MCF7, MDA-MB-231 and MDA-MB-157 cells were seeded and incubated with an ibidi culture insert until they reached subconfluent state and then transfected with PCDNA3, CMV-CYP2E1 or CYP2E1 shRNA plasmids and incubated for further 16 h. The size of the gap was measured after 16 h (MCF7, MDA-MB-157) and 8 h (MDA-MB-231). **(B)** Figure represents the calculations of migration distances of cells in **(A)**. Asterisks indicate significant difference at *P* <0.01. CYP2E1, cytochrome P450 E1; shRNA, short hairpin rRNA.

MCF7 cells overexpressing CYP2E1 displayed reduced migration capacity compared to those transfected with the empty vector (Figure 4A and B compare bar 1 to bar 4). On the other hand, MCF7 cells in which CYP2E1 had been silenced exhibited increased cell migration compared to MCF7 cells transfected with PCDNA3 (Figure 4A and B compare bar 1 to bar 7). Increased migration capacity was also observed in MDA-MB-231 cells in which CYP2E1 had been silenced (Figure 4A and B compare bar 2 to bar 8). CYP2E1 overexpression had marginal effect on the ability of MDA-MB-231 cells to migrate (Figure 4A and B compare bar 2 to bar 5). Overexpression or silencing of CYP2E1 in MDA-MB-157 cells did not change significantly the ability of these cells to migrate compared to those transfected with PCDNA3 (Figure 4A and B compare bar 3 to bars 6 and 9).

**Figure 5:**
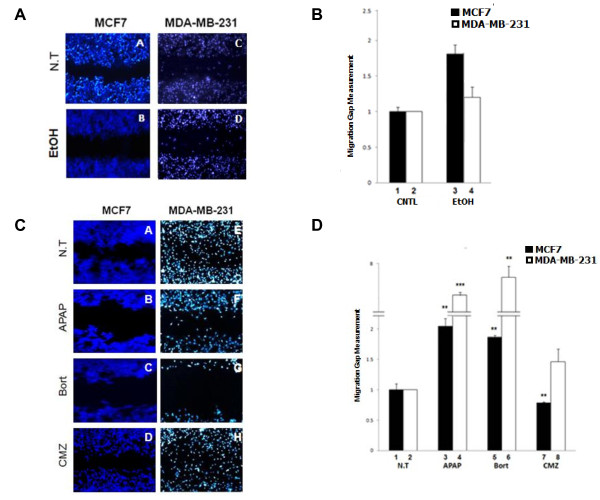
**CYP2E1 regulates cell migration in breast cancer cell lines. (A)** MCF7 and MDA-MB-231 cells were seeded and incubated with an ibidi culture insert up to subconfluent stage. Cell migration was estimated 16 h (MCF7) and 3 h (MDA-MB-231) after the addition of ethanol. **(B)** Figure represents the calculations of migration distances of cells in (A). **(C)** MCF7 and MDA-MB-231 cells were seeded and incubated as described in (A). Cell migration was estimated 3 h after the addition of acetaminophen (APAP), 24 h (MCF7) and 8 h (MDA-MB-231) after the addition of bortezomib (Bort) and 16 h after the addition of chlormethiazole (CMZ). **(D)** Figure represents the calculations of migration distances of cells in (C). Asterisks indicate significant difference at *P* <0.05 (2) and *P* <0.01 (3). CYP2E1, cytochrome P450 E1.

To further investigate the molecular mechanisms by which CYP2E1 gene expression affects cell migration in breast cancer cells, the migration capacity of untreated, ethanol, Bort, APAP [40] and the CYP2E1-specific inhibitor CMZ-treated [41] MCF7 and MDA-MB-231 cells was monitored. Ethanol induces CYP2E1 enzymatic activity, and Bort is a proteasome inhibitor that has been shown to induce response to ER stress by stimulating the accumulation of misfolded proteins in the ER [42]. In accord with results shown in Figure 4, decreased cell migration was observed in ethanol-treated MCF7 cells whereas induction of CYP2E1 by ethanol in MDA-MB-231 cells marginally decreased cell migration (Figure 5B, compare bar 3 to bar 1 and bar 4 to bar 2 respectively). Decreased cell migration was observed in APAP- and Bort-treated MCF7 and MDA-MB-231 compared to non-treated cells (Figure 5D, compare bars 3 and 5 to bar 1 and bars 4 and 6 to bar 2). This effect was not evident in MCF7 and MDA-MB-231 cells treated with CMZ compared to non-treated cells (Figure 5D, compare bars 7 and 8 to bars 1 and 2 respectively). Results presented in Figures 4 and 5 revealed that induction of CYP2E1 reduced the migration capacity whereas silencing of this CYP450 isoenzyme increased the number of MCF7 and MDA-MB-231 cells migrating within the gap indicating that CYP2E1 is potentially involved in the regulation of the migratory capacity of breast cancer cells.

### CYP2E1 gene expression is under p53 transcriptional control

The molecular mechanism underlying CYP2E1 induction has not been clearly elucidated but seems to involve messenger RNA or protein stabilization and/or transcriptional activation [43]. Taking into account the fact that the high levels of ROS produced in cells due to the function of this enzyme could stimulate the transcriptional activity of the cancer-related transcription factor p53 [44] as well as that decreased CYP2E1 levels observed throughout cancer progression in different cancers [19,45] led us to explore the possibility that CYP2E1 was a possible p53 transcriptional target.

To test the hypothesis whether higher CYP2E1 cellular levels in stage I breast tumors compared to stages II, III, and IV were a result of differential transcriptional regulation of its gene expression and potential involvement of p53 [46] in this process, we followed the CYP2E1 protein levels in five different breast cancer cells with different migratory potential and p53 status, namely MCF7 [20], T47D [20], MDA-MB-231 [21], MDA-MB-468 and MDA-MB-157 [20] exposed to either ethanol (MCF7, MDA-MB-231and MDA-MB-468) or etoposide (etop) (MCF7, MDA-MB-231, T47D and MDA-MB-157) treatment.

**Figure 6:**
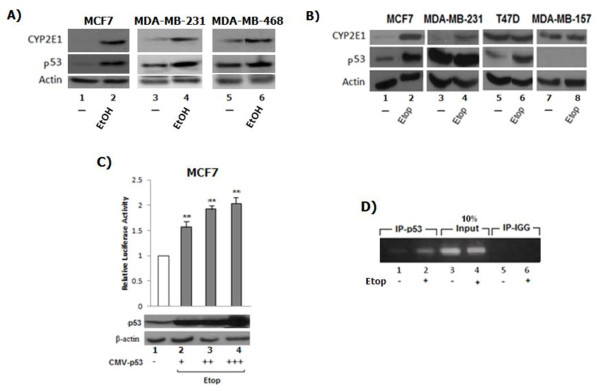
***CYP2E1 *gene expression is under the transcriptional control of p53. (A)** Breast cancer cell lines MCF7, MDA-MB-231, and MDA-MB-468 were treated with ethanol and cellular extracts were submitted to western blot analysis with anti-CYP2E1, anti-p53, and anti-β-actin antibodies. **(B)** MCF7, MDA-MB-231, T47D and MDA-MB-157 cells were treated with etoposide and total cellular extracts were subjected to western blot analysis as for (A). **(C)** CYP2E1-Luc reporter assay in MCF7 cells transfected with increasing amounts of CMV-p53. Data presented as fold induction of luciferase activity in cells. Results represent the average of three independent experiments. **(D)** Chromatin immuniprecipitated using specific p53 monoclonal antibody in non-treated or etoposide-treated MCF7 cells. The pulled-down DNA samples were amplified with PCR primers targeting CYP2E1 promoter. The resultant PCR product was then submitted to 1% agarose gel electrophoresis and visualised by UV light. CYP2E1, cytochrome P450 E1.

Accumulation of CYP2E1 protein was observed in response to ethanol in MCF7, MDA-MB-231 and MDA-MB-468 cells (Figure 6A, compare lanes 2, 4 and 6 to lanes 1, 3 and 5 respectively) whereas in response to etoposide treatment increased CYP2E1 protein levels were evident in MCF7 and MDA-MB-231 cells (Figure 6B, compare lanes 2 and 4 to lanes 1 and 3 respectively) and no significant changes in T47D and MDA-MB-157 cells (Figure 6B, compare lanes 6 and 8 to lanes 5 and 7 respectively). Reasons explaining the lack of correlation between the CYP2E1 and p53 protein levels in all cell lines could be that p53 is not the only transcription factor that mediates CYP2E1 gene expression and that p53 might be stabilised and accumulated in MDA-MB-231 and T47D cells but it is transcriptionally inactive.

To gain further insight in the transcriptional regulation of the *CYP2E1* gene expression and investigate the potential role of p53 in the regulation of the cellular levels of this enzyme the luciferase activity of the CYP2E1-Luc reporter containing the putative p53 binding sites [44] identified in the regulatory region of the promoter of this gene was studied in MCF-7 breast cancer cells. Gradual increase of ectopic expression of p53 resulted in upregulated CYP2E1-Luc reporter activity (Figure 6C, compare bars 2, 3 and 4 to bar 1). The p53 protein levels were analysed to confirm its ectopic overexpression (Figure 6B, compare lanes 2, 3 and 4 to lane 1). Furthermore, to confirm whether the putative p53 binding sites were functional, the recruitment of p53 to these putative binding sites was followed in control or treated with etoposide MCF7 breast cancer cells using ChIP assay. Increased occupancy of the CYP2E1 promoter by p53 in the etoposide-treated compared to the non-treated cells was observed (Figure 6D, compare lane 2 to lane 1) implying that p53 is possibly involved in the regulation of *CYP2E1* gene expression.

## Discussion

CYP2E1 is a drug-metabolising enzyme primarily expressed in hepatocytes, but has also been detected in other tissues including breast, brain, kidney and lungs [3]. CYP2E1 metabolises several pro-carcinogens including ethanol [7] and, for that reason, most of the research associated with CYP2E1 focuses on its role in liver diseases [8]. However, its functions in other tissues have not been extensively studied.

Clinical studies have indicated higher *CYP2E1* gene expression in breast tumours than normal tissues [47] and decreasing CYP2E1 levels as carcinogenesis progresses [19] suggesting that this enzyme might serve important functions in breast carcinogenesis. Given that CYP2E1 is one of the most active ROS-generating CYP450 isoforms [7] and considering the link between oxidative stress and tumour growth we hypothesised that CYP2E1-mediated ROS generation could regulate breast carcinogenesis.

In agreement with published observations [7], we detected increased intracellular ROS levels in breast cancer cells overexpressing CYP2E1 in a manner dependent on the status of p53. The p53 tumour suppressor plays an important role in the regulation of the cellular ROS generation and, in turn, ROS exert positive and negative effects on the p53 protein stability, transcriptional activity and gene target selectivity [46]. Low amounts of ROS correlate with stimulation of the expression of anti-oxidant p53 target genes (glutathione peroxidase 1 (GPX1) [48], aldehyde dehydrogenase 4 (ALDH4) [49], sestrins 1 and 2 (SESN1 and 2) [50]) and genes involved in the cellular energy metabolism such as SCO2 (synthesis of cytochrome c oxidase 2) [51], TIGAR (TP53-induced glycolysis and apoptosis regulator) [52] and PGM (phosphoglycerate mutase) [53]. The pro-oxidant effects of p53 are mediated by another set of its target genes such as the p53-induced gene 3 (PIG3) [54] the p66Shc [55] and the Bcl2 family members bax and PUMA [50,56]. The mechanisms directing the p53 target selectivity to antioxidant or pro-oxidant transcriptional target genes, thereby determining the final outcome of the p53-mediated cellular redox state, include posttranslational modifications and recognition of specific DNA binding sites in the regulatory regions of the promoters of pro-oxidant subsets of genes by domains different than the p53 DNA binding domain (proline-rich domain) [50,54,57] and the affinity of p53 binding to the promoter of its diverse transcriptional targets follows a hierarchical order that is dependent on the type of stress [22,58,59]. In these terms, the amount of CYP2E1-mediated ROS generation could induce either antioxidant or pro-oxidant p53 outcomes in a tissue and cell-type-dependent manner.

Since intracellular ROS levels are closely linked to the regulation of autophagy, we followed the autophagy biomarkers beclin-1, LC-3, Atg7 and Atg5 protein levels in breast cancer cells in which the CYP2E1 was either ectopically expressed or silenced. In line with potential tumour-suppressing function of autophagy, we identified that the protein levels of the autophagy markers [60,61], followed the same pattern as that of CYP2E1 in MDA-MB-231 cells. The role of p53 in linking cellular redox status energy metabolism and autophagy through transcription independent and transcription dependent mechanisms has been extensively investigated [62-64].

Taking into account the fact that CYP2E1 is predominantly localized in the endoplasmic reticulum [65] as well as the link between autophagy and ER stress [36,37] we investigated the possibility that CYP2E1 was part of the ER stress signalling transduction pathway. Integrated regulation of autophagy, ER stress and unfolded protein response determines the cell fate in breast cancer [61]. ER stress is able to both inhibit and activate the p53 pathway [66,67]. ER stress in MCF-7 cells stimulates p53 nuclear localization, transcriptional activity and protein stability through the NF-κB signalling pathway [68]. In accord with published evidence indicating that CYP2E1 is a positive regulator of UPR [21], our results lent support to the notion that CYP2E1 induces UPR in MCF7 breast cancer cells transfected with CYP2E1. UPR is altered in many types of cancer [69], including breast cancer, and in some cases contributes to chemoresistance [24,35,70,71], highlighting the importance of our findings for cancer therapy.

The decreasing levels of CYP2E1 in advanced stages of breast cancer cells [19] together with our observations indicating that high cellular levels of CYP2E1 induce autophagy in MDA-MB-231 cells and UPR in MCF7 cells, both of which are regulators of the tumour microenvironment [61], led us to investigate its role in regulating migration of breast cancer cells. To address this question, migration was followed in breast cancer cells expressing different levels of CYP2E1. Our results provided evidence that CYP2E1 expression in breast cancer cells plays a role in the determination of migratory capacity. In particular, CYP2E1 ectopic expression inhibited MCF7 and MDA-MB-231 cell migration whereas CYP2E1 silencing or inhibition of its enzymatic activity promoted the ability of these cells to migrate. Differences in the p53 status between MDA-MB-231 and MDA-MB-157 cells, as well as potential defects in the phosphorylation events stabilising microtubule-associated proteins in a manner involving CYP2E1-mediated ROS, could explain the difference in terms of metastasis in the two cell lines. Cytoskeletal alterations mediated by microtubule-associated proteins such as members of the tubulin family [72] and the microtubule-associated protein Tau [73] play an important role in breast cancer cells’ metastasis and their cellular levels are affected by p53 [72] and the estrogen receptor [74,75] status. Another possible mechanism explaining the effects of the p53-CYP2E1-ROS generation axis on cell invasion and metastasis could be mediated by heparanase, which is a p53 transcriptional target [76] and is involved in the regulation of the ER stress-mediated breast cancer cell migration [77], but this hypothesis requires experimental validation.

To investigate the factors regulating *CYP2E1* gene expression, its protein levels were followed in breast cancer cells with different p53 and estrogen receptor status treated with either ethanol, which induces CYP2E1 enzymatic activity, or the topoisomerase II inhibitor etoposide, which induces DNA damage and activates p53 response. Our results strengthened the hypothesis that p53 is a possible upstream regulator of *CYP2E1* gene expression.

Taken together, the results presented in this manuscript provide evidence to suggest that CYP2E1 plays an important role in breast carcinogenesis and the extent of the oxidative stress mediated by CYP2E1 determines distinct p53-mediated effects on autophagy, ER stress and metastasis in breast cancer cells. Since CYP2E1 overexpression restrains migration in the invasive MDA-MB-231 cells, manipulation of CYP2E1 cellular levels could potentially be beneficial for better outcome of late stages of breast cancer. In addition, since this enzyme is involved in the metabolism of alcohol, observations described in this manuscript could be an additional link between chronic alcohol consumption and breast cancer [78,79].

## Conclusions

Ectopic expression of CYP2E1 in breast cancer cells increase ROS generation, modulates autophagy and regulates ER stress and unfolded protein response in a cell-type-dependent manner. In addition, induction of *CYP2E1* gene expression is under the transcriptional control of the p53 tumor suppressor and its activation inhibits migration of the highly invasive MDA-MB-231 breast cancer cells. The results shown in this manuscript suggest that manipulation of CYP2E1 protein levels and enzymatic activity could be potentially exploited in breast cancer therapy.

## Abbreviations

APAP: Acetaminophen; ATF6: Activating transcription factor 6; ATGs: Autophagy-regulated genes; Bort: Bortezomib; ChIP: Chromatin immunoprecipitation; CHOP: CCAAT/enhancer-binding protein (C/EBP) homologous protein; CMZ: Chlormethiazole; CYP2E1: Cytochrome P450 E1; DAPI: 4′,6-Diamidino-2-phenylindole; DDT: Dichlorodiphenyltrichloroethane; ECACC: European collection of cell cultures; EDTA: Ethylenediaminetetraacetic acid; ER: Endoplasmic reticulum; FACS: Fluorescence-activated cell sorting; GRP78: Glucose-regulated protein 78; H2DCFDA: 2 2.9.2 2′,7′-dichlorodihydrofluorescein diacetate; IRE1: Inositol-requiring enzyme 1; LC3: Light chain 3; NAC: N-acetylcysteine; NASH: Non-alcoholic steatohepatitis; PBS: Phosphate-buffered saline; PERK: PKR-like endoplasmic reticulum kinase; PMSF: Phenylmethylsulphonyl fluoride; ROS: Reactive oxygen species; shRNA: Short hairpin RNA; UPR: Unfolded protein response; XBP1: Xbox binding protein 1.

## Competing interests

The authors declare that they have no competing interests.

## Authors’ contributions

TL carried out the construction of the CYP2E1 luciferase reporter and the shRNA engineering, the measurement of ROS levels, the western blot analysis, the luciferase reporter, the chromatin immunoprecipitation, the scratch wound assays, performed statistical analysis and drafted the manuscript. RR contributed to the FACS analysis for the measurement of ROS levels, and the chromatin immunoprecipitation, helped to draft the manuscript and critically revised it. SS performed western blot experiments to address the referees’ comments. RG performed the ROS measurements to address the referees’ comments. MKD participated in the design of the study, the interpretation of the data, helped to draft the manuscript and critically revised it. CD conceived the study, participated in the design of the experiments, interpreted the data, coordinated the research and drafted the final manuscript. All authors read and approved the final manuscript.
